# The interaction of fat mass and obesity associated gene polymorphisms and dietary fiber intake in relation to obesity phenotypes

**DOI:** 10.1038/s41598-017-18386-8

**Published:** 2017-12-22

**Authors:** Firoozeh Hosseini-Esfahani, Gelareh Koochakpoor, Maryam S. Daneshpour, Parvin Mirmiran, Bahareh Sedaghati-khayat, Fereidoun Azizi

**Affiliations:** 1 Nutrition and Endocrine Research Centre, Research Institute for Endocrine Sciences, Shahid Beheshti University of Medical Sciences, Tehran, Iran; 2 Maragheh University of Medical Sciences, Maragheh, Iran; 3 Cellular and Molecular Endocrine Research Centre, Research Institute for Endocrine Sciences, Shahid Beheshti University of Medical Sciences, Tehran, Iran; 4 Faculty of Nutrition Sciences and Food Technology, National Nutrition and Food Technology Research Institute, Shahid Beheshti University of Medical Sciences, Tehran, Iran; 5 Endocrine Research Centre, Research Institute for Endocrine Sciences, Shahid Beheshti University of Medical Sciences, Tehran, Iran

## Abstract

Controversies surrounding the effectiveness of fiber intake for prevention of obesity can be attributed to differences in the genetic makeup of individuals. This study aims to examining the interaction between dietary fiber intake and common fat mass and obesity–associated (*FTO*) single-nucleotide polymorphisms (SNPs), in relation to obesity. Subjects of this nested case-control study were selected from among adult participants of the Tehran Lipid and Glucose Study. Cases (n = 627) were individually matched with controls, who had normal weight. Six selected SNPs (rs1421085, rs1121980, rs17817449, rs8050136, rs9939973, and rs3751812) were genotyped by tetra-refractory mutation system-polymerase chain reaction analysis. Genetic risk scores (GRS) were calculated using the weighted method. A significant interaction was observed between total fiber intake and the GRS in relation to obesity (Pinteraction = 0.01); the difference in the risk for obesity was more pronounced in individuals with GRS ≥ 6 who consumed ≥ 14 grams of fiber a day (OR: 2.74, CI: 2.40–3.35 vs Ref.; P trend = 0.0005) than in individuals with GRS < 6 (P trend = 0.34). Dietary fiber intakes modified the association of *FTO* SNPs and the GRS with general obesity, an effect which was more pronounced in those who consumed high levels of dietary fiber and had a high number of risk alleles.

## Introduction

Obesity, a fast growing major health problem today, is a leading cause of death worldwide due to the increased risk of many chronic diseases in persons with obesity. Genetic analyses have shown that genetic factors contribute to around 40–70% of the population variation in body mass index (BMI)^1,2^. Of these factors, the fat mass and obesity–associated (*FTO*) gene is now recognized as the strongest common genetic predictor of obesity^3,4^. It is located in chromosome region 16q12.2 and is highly expressed in the hypothalamus nuclei, which govern food intake and appetite^5^. Consequently, *FTO* single-nucleotide polymorphisms (SNPs) increase the risk of obesity by 1.20–1.32 fold in Europeans^6^ and by 1.25 fold in Asians^7^. However, genetic etiology alone cannot explain the rapid rises in the prevalence of obesity in recent years, because gene pools of specific populations have been relatively constant for many generations^8^.

Dramatic changes in environmental factors, such as lifestyle and dietary habits, are believed to play a role in the rising trend of obesity. A high-fiber diet is one factor that has received substantial attention in obesity prevention, with some studies reporting that increasing dietary fiber can reduce the likelihood of weight gain^9–11^, although contradictory results are noted^12^. Several clinical intervention trials have shown weight reduction associated with diets rich in dietary fiber or fiber supplements^13,14^, whereas other studies have failed to demonstrate any effect^15–17^. These controversies on the effectiveness of fiber intake for prevention of obesity could be attributed to differences in people’s genetic structure, indicating that the risk of obesity is more likely determined by gene-environmental factor interactions than by dietary fiber alone.

Genome wide association studies have indicated that each identified obesity locus explains only a small fraction of the observed variations in BMI. A strategy that combines multiple genetic variants related to obesity into a genetic risk score (GRS) therefore might improve the identification of individuals at risk of developing obesity. Few studies have examined the interaction of dietary factors (e.g., fiber intake) and *FTO*-genetic predisposition or genetic structure on the risk of obesity in a Middle-Eastern population, and most studies to date pertain to interactions between *FTO* and dietary macronutrients. The objective of the present nested case-control study was to examine the hypothesis that dietary fiber could interact with *FTO* SNPs (rs1121980, rs1421085, rs9939973, rs8050136, rs17817449, and rs3751812), singly and in combination, in relation to obesity phenotypes among adults.

## Results

General characteristics of participants (cases and controls) are shown in Table 1. The two groups showed no significant differences in terms of physical activity, smoking, nutrient intakes; however, years of educational attainment and energy intake differed significantly among the cases and controls. The effect size and risk allele of each SNP for obesity traits are shown in Table 2. Frequencies of genotypes or alleles also did not differ between the two groups. Genotype frequencies were in Hardy Weinberg equilibrium (P > 0.2). The median of GRS among the participants was 6.

**Table 1:** Characteristics of the study population in cases and controls^†^ (Tehran Lipid and Glucose Study).

	Normal BMI^a,b^ (n = 627)		Obese^c^ (n = 627)	
		SD		SD
**Baseline Age (y)** ^**c**^				
Men	34.01	11	34.25	11
Women	34.82	11	34.91	10
Current smokers (%)	16.7		13.9	
Low physical activity (%)	40.2		41.3	
Education level ≥ 14 years (%)	27.3		20.4^*^	
Baseline BMI (Kg/m^2^)	21.54		25.03^*^	
Baseline WC^d^(cm)	73.54	16	82.25^*^	23
Energy intake (KJ/day)	9959	4094	10612^*^	4416
Carbohydrate (% of energy)	58.07	6	58.08	6
Total fiber intake (g/4186 KJ)	18.06	6	18.57	6
Protein intake (% of energy)	14.46	3	14.44	2
Total fat (% of energy)	30.9	6	30.7	6
Saturated fat (% of energy)	10.25	3	10.07	2
MUFA (% of energy)^e^	10.51	2	10.41	2
PUFA (% of energy)^f^	6.35	2	6.32	2

^a^BMI: body mass index. ^b^BMI from 18.5 up to 25 kg/m^2^,^c^BMI ≥ 30 kg/m^2^. ^d^WC: waist circumference. ^e^MUFA, Mono-unsaturated fatty acids. ^f^PUFA, Poly-unsaturated fatty acids. *P < 0.05; ^†^values are mean unless otherwise listed.

**Table 2:** Allele, genotype frequency and risk alleles of *FTO* SNPs in cases (obese) and controls (non-obese): Tehran Lipid and Glucose Study.

Frequency	Normal BMI^a^ (n = 627)	Obese^b^ (n = 627)	Risk allele^c^	Effect size (odds ratio)
**rs1121980**				1.34
Allele	A: 38 (463) ^C^	A: 39(474)	A	
	G: 62(755)	G: 61(752)		
Genotype	AA: 15(92)	AA: 13(77)		
	GA: 46(279)	GA: 52(320)		
	GG: 39(238)	GG: 35(216)		
**rs1421085**				1.43
Allele	C: 37(458)	C: 36(446)	C	
	T: 63(770)	T: 64(792)		
Genotype	CC:14(87)	CC:12(72)		
	TC: 44(272)	TC: 51(314)		
	TT: 42(260)	TT: 37(228)		
**rs9939973**				1.10
Allele	A: 37.9 (468)	A: 38.2 (469)	A	
	G: 62.1 (768)	G: 61.8 (759)		
Genotype	AA: 15(93)	AA: 12.2(76)		
	GA: 45.6(282)	GA: 52(319)		
	GG: 39.3(243)	GG: 35.8(220)		
**rs8050136**				1.25
Allele	A:34(417)	A: 35(435)	A	
	G: 66(823)	G: 65(795)		
Genotype	AA: 13(78)	AA: 11(66)		
	GA: 42(261)	GA: 49(303)		
	GG: 45(281)	GG: 40(246)		
**rs17817449**				1.54
Allele	G: 33(413)	G: 36(444)	G	
	T: 67(832)	T: 64(802)		
Genotype	GG: 12(75)	GG: 11(68)		
	TG: 42(264)	TG: 49(308)		
	TT: 46(284)	TT: 40(247)		
**rs3751812**				1.52
Allele	G: 67(829)	G: 65(799)	T	
	T: 33(405)	T: 35(427)		
Genotype	GG: 47(290)	GG: 40(247)		
	GT: 40(249)	GT: 50(305)		
	TT: 13(78)	TT: 10(61)		

^a^BMI (body mass index): 18.5–25 kg/m^2, b^BMI ≥ 30 kg/m^2^, ^C^% (n)

All allele frequencies were in Hardy-Weinberg equilibrium (P > 0.2), except allele frequency of rs9939973 in obese participants (P = 0.01).

^c^Risk allele based on data from GWAS catalog (The NHGRI-EBI Catalog of published genome-wide association studies) and the Phenotype-Genotype Integrator^44,45^.

Effect sizes were derived from the previous literature and reported meta-analysis^46,49,52^.

### Interactions of SNPs and dietary fiber intake in relation to obesity

Dietary fiber modulated the association of obesity with genotype groups for three SNPs– rs8050136 (P interaction = 0.02), rs17817449 (P interaction = 0.02) and rs3751812 (P interaction = 0.01) after adjustment for energy intake and years of education. Risk allele carriers of these SNPs benefited more from dietary fiber than the other genotypes. No similar significant interaction was observed in relation to obesity between dietary fiber and the other SNPs (rs1121980, rs1421085, and rs9939973) examined in this study (Table 3).

**Table 3:** Adjusted ORs (95%CI) for obesity according to quartiles of dietary fiber intake and *FTO* SNP genotypes^a^ (Tehran Lipid and Glucose Study).

	Q1	Q2	Q3	Q4	P for trend	P for interaction
**rs1121980**						0.15
GG	0.96 (0.54–1.69)	0.90 (0.53–1.54)	0.85 (0.50–1.47)	1	0.36	
GA + AA	1.47 (1.02–2.03)	1.14 (0.75–1.83)	1.05 (0.65–1.71)	0.58 (0.11–1.17)	0.03	
**rs1421085**						0.85
TT	0.82 (0.47–1.42)	0.88 (0.52–1.49)	0.79 (0.46–1.34)	1	0.27	
TC + CC	1.37 (0.83–2.18)	1.14 (0.52–1.81)	1.00 (0.51–1.68)	0.73 (0.51–1.39)	0.06	
**rs9939973**						0.70
GG	0.91 (0.52–1.58)	0.86 (0.50–1.46)	0.83 (0.49–1.43)	1	0.22	
GA + AA	1.59 (1.12–2.16)	1.15 (0.70–1.77)	1.02 (0.63–1.65)	0.65 (0.23–1.37)	0.05	
**rs8050136**						
GG	0.85 (0.50–1.43)	0.75 (0.45–1.25)	0.98 (0.61–1.58)	1	0.25	0.02
GA + AA	1.95 (1.57–2.54)	1.21 (0.91–1.93)	0.90 (0.57–1.52)	0.62 (0.21–1.12)	0.009	
**rs1781749**						
TT	0.79 (0.47–1.32)	0.76 (0.46–1.27)	0.62 (0.37–1.04)	1	0.21	0.02
TG + GG	1.88 (1.65–2.37)	1.17 (0.83–1.87)	0.79 (0.47–1.31)	0.41 (0.15–0.97)	0.001	
**rs3751812**						
GG	0.80 (0.48–1.35)	0.71 (0.43–1.19)	0.61 (0.37–1.02)	1	0.36	0.01
GT + TT	1.65 (1.27–2.22)	1.25 (0.68–1.73)	1.24 (0.67–1.73)	0.32 (0.11–0.94)	0.03	

OR: Odds Ratio, Q: Quartiles of dietary fiber intake (Q1: < 7.06, Q2: 7.06–9.13, Q3: 9.14–11.26, Q4 > 11.26 g/4186 KJ), *FTO*: Fat mass and Obesity associated gene, SNP: Single Nucleotide Polymorphism. ^a^ORs (95% CI) were calculated using conditional logistic regression model, adjusted for education level and energy intake. Participants were classified (8 groups) according to quartiles of dietary fiber intake and genotypes. The highest quartile of dietary fiber intake and homozygote genotype of major allele was used as the reference group.

### Interactions of SNPs and dietary fiber intake in relation to abdominal obesity

Dietary fiber only modulated the association of risk allele carriers (TG + TT) of rs3751812 with abdominal obesity (P interaction = 0.01). In T allele carriers, the risk of abdominal obesity decreased across quartiles of dietary fiber (P trend = 0.003), although this association was not significant in GG homozygote carriers (P trend = 0.62) (Table 4).

**Table 4:** Adjusted ORs (95%CI) for abdominal obesity according to quartiles of dietary fiber intake and *FTO* SNP genotypes (Tehran Lipid and Glucose Study).

	Q1	Q2	Q3	Q4	P for trend	P for interaction
**rs1121980**						0.58
GG	0.80 (0.45–1.44)	0.94 (0.54–1.64)	0.93 (0.54–1.61)	1	0.36	
GA + AA	0.93 (0.57–1.52)	0.82 (0.50–1.33)	0.74 (0.46–1.21)	0.52 (0.10–1.17)	0.15	
**rs1421085**						0.81
TT	0.95 (0.55–1.63)	0.96 (0.56–1.64)	1.01 (0.59–1.72)	1	0.76	
TC + CC	0.90 (0.55–1.46)	0.84 (0.52–1.36)	0.74 (0.46–1.19)	0.77 (0.48–1.25)	0.63	
**rs9939973**						0.66
GG	0.85 (0.49–1.49)	0.98 (0.56–1.70)	0.94 (0.55–1.61)	1	0.88	
GA + AA	0.96 (0.59–1.55)	0.85 (0.53–1.38)	0.76 (0.47–1.23)	0.76 (0.47–1.24)	0.47	
**rs8050136**						
GG	0.95 (0.56–1.60)	1.12 (0.66–1.89)	1.17 (0.70–1.96)	1	0.61	0.37
GA + AA	1.55 (1.17–2.21)	0.90 (0.56–1.44)	0.75 (0.46–1.20)	0.62 (0.21–1.12)	0.01	
**rs1781749**						
TT	0.96 (0.57–1.62)	1.10 (0.65–1.86)	1.17 (0.75–1.96)	1	0.55	0.42
TG + GG	1.51 (1.19–2.15)	0.87 (0.83–1.87)	0.72 (0.47–1.31)	0.70 (0.44–1.29)	0.02	
**rs3751812**						
GG	0.99 (0.59–1.66)	1.18 (0.70–1.99)	1.19 (0.71–1.91)	1	0.62	0.01
GT + TT	1.98 (1.63–2.67)	0.88 (0.55–1.43)	0.70 (0.44–1.13)	0.51 (0.28–1.17)	0.003	

OR: Odds Ratio, Q: Quartiles of dietary fiber intake (Q1: < 7.06, Q2: 7.06–9.13, Q3: 9.14–11.26, Q4: > 11.26 g/4186 KJ), *FTO*: Fat mass and Obesity associated gene, SNP: Single Nucleotide Polymorphism. ^a^ORs (95% CI) were calculated using conditional logistic regression model, adjusted for education level, age, gender, smoking status, physical activity and energy intake. Participants were classified (8 groups) according to quartiles of dietary fiber intake and genotypes. The highest quartile of dietary fiber intake and homozygote genotype of major allele was used as the reference group.

The ORs of high waist to hip ratio differed across quartiles of dietary fiber intake in the risk allele carriers of rs3751812. Those in the lowest quartile of dietary fiber had an over than two-fold OR for abdominal obesity compared with those in the highest quartile (P trend = 0.001, P interaction = 0.01), while ORs of a high waist to hip ratio did not differ across the quartiles of dietary fiber in the GG genotype group (Table 5).

**Table 5:** Adjusted ORs (95%CI) for high waist to hip ratio (WHR) according to quartiles of dietary fiber intake and *FTO* SNP genotypes (Tehran Lipid and Glucose Study).

	Q1	Q2	Q3	Q4	P for trend	P for interaction
**rs1121980**						0.13
GG	2.02 (0.88–4.66)	2.46 (1.09–5.54)	2.50 (1.10–5.68)	1	0.06	
GA + AA	1.69 (0.78–3.63)	1.77 (0.82–3.80)	1.81 (0.84–3.89)	1.35 (0.59–3.04)	0.74	
**rs1421085**						0.54
TT	2.05 (0.93–4.53)	2.47 (1.13–5.39)	2.54 (1.16–5.58)	1	0.77	
TC + CC	1.59 (0.75–3.37)	1.71 (0.81–3.61)	1.77 (0.84–3.74)	1.35 (0.61–2.99)	0.88	
**rs9939973**						0.12
GG	1.96 (0.87–4.38)	2.37 (1.08–5.21)	2.44 (1.10–5.39)	1	0.08	
GA + AA	1.56 (0.76–3.28)	1.71 (0.81–3.59)	1.71 (0.81–3.60)	1.23 (0.55–2.74)	0.72	
**rs8050136**						
GG	1.72 (0.81–3.62)	2.33 (1.12–4.83)	1.85 (0.88–3.89)	1	0.47	0.61
GA + AA	1.38 (0.67–2.84)	1.41 (0.69–2.88)	1.71 (0.84–3.48)	1.11 (0.51–2.41)	0.52	
**rs1781749**						
TT	1.75 (0.83–3.68)	2.32 (1.12–4.80)	1.97 (0.94–4.13)	1	0.58	0.60
TG + GG	1.40 (0.68–2.87)	1.40 (0.68–2.86)	1.69 (0.83–3.45)	1.15 (0.53–2.46)	0.86	
**rs3751812**						
GG	1.89 (0.88–4.03)	1.58 (0.76–3.30)	1.52 (0.72.3.17)	1	0.62	0.01
GT + TT	2.73 (1.45–5.41)	2.17 (0.95–4.28)	1.79 (0.86.3.72)	1.21 (0.55–2.67)	0.001	

OR: Odds Ratio, Q: Quartiles of dietary fiber intake (Q1: < 7.06, Q2:7.06–9.13, Q3:9.14–11.26, Q4 > 11.26 g/4186 KJ), *FTO*: Fat mass and Obesity associated gene, SNP: Single Nucleotide Polymorphism. ^a^ORs (95% CI) were calculated using conditional logistic regression model, adjusted for education level, age, gender, smoking status, physical activity and energy intake. Participants were classified (8 groups) according to quartiles of dietary fiber intake and genotypes. The highest quartile of dietary fiber intake and homozygote genotype of major allele was used as the reference group.

### Interactions of obesity genetic risk score and dietary fiber intake in relation to obesity and abdominal obesity

Significant interactions were observed between dietary fiber and the GRS in relation to obesity (P interaction = 0.01). The difference in the risk for obesity was more pronounced in individuals with GRS ≥ 6 who were in the highest quartile of dietary fibers, compared to the lowest quartile (OR Q4:0.67, CI:0.41–1.10; OR Q1:1.83, CI:1.32–2.54; P trend = 0.004), than in individuals with GRS < 6 (P trend = 0.71). The difference in the risk for obesity was also more pronounced in individuals with GRS ≥ 6, and those who consumed ≥ 14 grams of fiber/day (P trend = 0.0005) than in individuals with GRS < 6 (P trend = 0.34) (Table 6).

**Table 6:** Adjusted ORs (95%CI) for obesity and abdominal obesity according to categories of dietary fiber intake and GRS^a^.

	Quartiles of dietary fiber intake						Low fiber intake	High fiber intake	P for trend	P for interaction
	Q1	Q2	Q3	Q4	P for trend	P for interaction	< 14 gr	≥ 14 gr		
**Obesity**						0.01				0.004
GRS ≥ 6	1.83 (1.32–2.54)	1.30 (0.81–2.07)	0.80 (0.49–1.32)	0.67 (0.41–1.10)	0.004		2.74 (2.40–3.35)	1	0.0005	
GRS < 6	1.27 (0.80–2.01)	0.82 (0.52–1.31)	0.80 (0.49–1.30)	1	0.71		0.64 (0.29–1.45)	0.55 (0.29–1.02)	0.34	
**Abdominal obesity**						0.38				0.83
GRS ≥ 6	1.00 (0.62–1.62)	0.88 (0.54–1.37)	0.79 (0.46–1.32)	0.70 (0.44–1.12)	0.07		1.13	1	0.55	
GRS < 6	0.99 (0.60–1.64)	1.10 (0.66–1.82)	1.23 (0.75–2.02)	1	0.68		0.98 (0.69–1.38)	0.89 (0.56–1.40)	0.70	
**High WHR**						0.14				0.06
GRS ≥6	1.29 (0.63–2.64)	1.41 (0.54–1.37)	1.68 (0.83–3.37)	1.13 (0.53–2.40)	0.93		0.86 (0.14–2.15)	1	0.70	
GRS < 6	1.84 (0.90–3.77)	2.12 (1.05–4.30)	1.86 (0.91–3.81)	1	0.06		2.45 (1.64–3.94)	2.13 (1.21–3.75	0.03	

OR: Odds Ratio, Q: Quartiles of dietary fiber intake (Q1: < 7.06, Q2: 7.06–9.13, Q3: 9.14–11.26, Q4 > 11.26 g/4186 KJ), GRS: Genetic risk score, was calculated on the basis of the 6 selected single nucleotide polymorphisms of fat mass and obesity associated gene (FTO) using a weighted method. ORs (95% CI) were calculated using conditional logistic regression model, adjusted for education level and energy intake. Participants were classified (8 groups) according to quartiles of dietary fiber intake and GRS. The highest quartile of dietary fiber intake and GRS < 6 was used as the reference group; also participants were classified (4 groups) according to low and high fiber intake and GRS. The high fiber intake and GRS > 6 was used as the reference group.

## Discussion

Our study showed that dietary fiber could modify the association of *FTO* SNPs and the genetic risk score with general obesity, an effect that was more pronounced in subjects who consumed high levels of dietary fiber (≥14 gr/day) and had a high genetic risk score, since they had the lowest risk of obesity when compared to individuals with a low dietary fiber and a low genetic risk score. The only significant gene-fiber interaction in relation to abdominal obesity was observed between fiber and rs3751812 in relation to high WC and high WHR. a result suggesting that individuals with high number of risk alleles could benefit more from a high dietary fiber than individuals with low number of risk alleles. Our findings on the *FTO* gene–fiber interaction therefore could have significant implications in public health for the prevention and management of obesity.

This is the first study to examine a possible *FTO*–dietary fiber interaction in relation to obesity phenotypes in a Middle-Eastern population. Findings of Villegas *et al*. suggest that the modifiable effect of dietary fiber on the association of genotype-phenotype may differ by race or ethnicity; they found that dietary fiber modified the association between *FTO* rs8050136 and diabetes in non-Hispanic whites, whereas no significant interaction term was observed consistently across non-Hispanic blacks and Mexican American participants^18^.

Examination of the *FTO*–diet interaction on obesity-related traits in an Asian Indian population showed that dietary fiber modified the association of the *FTO* SNP rs11076023 and waist circumference (WC), indicating that this effect was more pronounced on central obesity than on general obesity^19^. On the contrary, a study by Lappalainen *et al*. showed that the *FTO* (rs9939609)–fiber interactions did not reach conventional statistical significance in relation to BMI^20,21^, although the association between *FTO* and BMI was more pronounced in those having a diet high in fat and low in carbohydrates and fiber^20^.

Previous reports of *FTO*-diet interactions demonstrate that the *FTO* effect on obesity may be modulated by a healthy dietary pattern. Adherence to the traditional Mediterranean dietary pattern, which is low in saturated fat and includes foods rich in fiber (including vegetables, fruits, legumes, and nuts), fish, and olive oil showed interactions with the *FTO*-rs9939609. A low adherence to the Mediterranean diet resulting higher type 2 diabetes risk in risk allele carriers (OR = 1.21, 95%CI: 1.03–1.40; P = 0.019 for *FTO*-rs9939609) when compared to the wild-type (non-risk allele) subjects^22^. A three year follow-up of individuals adhering to a Mediterranean style diet revealed a lower weight gain in the risk allele carriers despite the lack of any gene-nutrient interaction^23^.

The interaction of *FTO* SNPs and consumption of unhealthy food groups (e.g. salty snacks, sweets, and fried foods), which were typically energy dense with limited dietary fiber and nutritional value, was analyzed in other studies in relation to obesity. Findings demonstrated that subjects with higher number of *FTO* risk alleles and with the highest intake of fried foods and sugary beverages might be more susceptible to obesity than individuals with lower consumption of unhealthy food groups^24^, indicating that encouraging the consumption of low energy density and high fiber foods may be an effective public health strategy to avoid excessive fat accumulation and that *FTO* risk allele carriers could benefit more from dietary guidelines aimed at increasing dietary fiber intake.

Many studies indicate that dietary fiber induces greater satiety when compared with simple sugars^25,26^; this increased satiety may result from several factors: the innate physical properties of dietary fiber (bulking, gel formation, and alteration of the viscosity of the gastric contents)^27^, delaying gastric emptying and subsequent gastric distention, blunting of postprandial glucose and insulin responses, and the effects on secretion of gut peptide hormones that regulate satiation (such as ghrelin, cholecystokinin, peptide YY, and glucose-dependent insulinotropic peptide)^28^. Dietary fiber also affects the expression of the gastric ghrelin gene, as confirmed in a previous study^29^.

Fermentable fibers are consumed by the intestinal bacteria, which produce short-chain fatty acids, including acetate, propionate, and butyrate, which impact the expression of many genes^30–32^. Histone tail acetylation is believed to limit the accessibility of a gene to the transcription machinery^33^; therefore, inhibition of histone deacetylase by butyrate may contribute to increased mRNA expression of several genes, such as PPAR-δ (peroxisome proliferator-activated receptor-δ). The binding of PPARs to specific DNA sequences, called PPAR response elements, around target genes then activates DNA transcription and gene expression^34^. The role of PPARs in gene regulation in adipose tissue is not clear, but our results propose that fiber intake, as an activator of PPAR-δ, may change the expression of *FTO* gene, a hypothesis which requires further molecular studies for confirmation.

The strengths of our study include its prospective design and use of repeated measures of dietary fiber intake and BMI that minimized the potential reverse causality that is more likely to occur in cross-sectional studies. Using subjects matched individually by age and sex and conducting extensive adjustment for potential confounders were the other strengths. Nevertheless, several limitations need to be acknowledged. Measurement errors of dietary fiber were inevitable, although the food-frequency questionnaire (FFQ) had been extensively validated. Our study population was highly homogeneous, as the study included only residents of district 13 of Tehran; its genetic-predisposition score therefore captured only a small amount of variation in BMI.

In conclusion, our findings provide evidence for an association between higher consumption of dietary fiber and a lower risk of general obesity in at least those people susceptible to obesity. The only rs3751812 × fiber interaction was found in relation to abdominal obesity which highlights beneficial effects of dietary fiber for individuals with 1 or 2 rs3751812 risk alleles.

## Materials and Methods

### Study population

Subjects of this nested case-control study were selected from participants of the Tehran Lipid and Glucose Study (TLGS), a large-scale, community-based, prospective study being performed on a sample of residents of district 13 of Tehran, capital of Iran. The first phase of the TLGS was conducted from 1999 to 2001 on 15 005 subjects, aged ≥ 3 years, and follow-up examinations have been conducted every 3 years (2002–2005; 2006–2008; 2008–2011, and 2011–2014) to identify newly developed diseases. Details of this ongoing cohort study have been published elsewhere^35,36^.

Of 11 001 and 9807 individuals, aged ≥ 18 years who participated in baseline and second follow-up surveys, respectively, 1813 subjects were excluded because they were evaluated as obese at either baseline or the second follow-up survey. In the current study, 1000 cases were randomly selected from among participants who developed obesity in the third (n = 528), fourth (n = 416), or the fifth (n = 286) phases. Individuals with a history of weight loss or gain >5 kg in the last 6 months, those who were pregnant and lactating, or those who had taken drugs that affect weight were excluded from the study, leaving 880 cases included in the study. Each of these 880 cases was individually pair matched by age (±5 years) and sex with a random control from a population with normal weight at the time that the corresponding case developed obesity. Cases/controls lacking DNA purification in the range of 1.7 < A260/A280 < 2, and those whose reported energy intakes divided by the predicted energy intake did not qualify for the ± 3 SD range, were excluded, eventually data from 627 pairs of persons with obesity and their matched controls (1254 individuals) were ultimately analyzed.

Written informed Consent was obtained from all participants. The study was conducted in accordance with the Declaration of Helsinki and the study protocol was approved by the ethical committee of the Research Institute for Endocrine Sciences, Shahid Beheshti University of Medical Sciences, Tehran, Iran.

### Measurements

Dietary intake was assessed using a valid and reliable 168-item semi-quantitative FFQ to assess the usual food intakes of individuals during the 12 months before the examination^37,38^. The consumption frequency of each food item on a daily, weekly, or monthly basis was converted to daily intakes, and the portion sizes were then converted to grams using measuring cups and spoons. The Iranian food composition table (FCT) is incomplete; therefore, we used the United States Department of Agriculture (USDA) FCT to analyze foods^39^. However, the Iranian FCT was used for some national foods and beverages when these were not listed in the USDA FCT^40^.

The body weight of each participant was measured to the nearest 100 g using digital scales while the subjects were minimally clothed and not wearing shoes. Height was measured to the nearest 0.5 cm with a tape measure while the subjects were in a standing position, with their shoulders in a normal alignment and with shoes removed. Circumferences were measured to the nearest millimeter using a flexible tape. WC was taken at the end of normal expiration, over light clothing, with the unstretched tape meter positioned at the level of umbilicus, without exerting any pressure on the body surface; measurements were recorded to the nearest 0.1 cm. Hip circumference was measured at the level of maximal protrusion of the gluteal muscles. Waist to hip ratio (WHR) was calculated as WC (cm) divided by hip circumference (cm). Physical activity level was assessed with high reliability and relatively moderate validity using the Persian translated modifiable activity questionnaire (MAQ)^35^. The frequency and time spent on light, moderate, hard, and very hard intensity activities, according to the list of common activities of daily life over the past year, were obtained, and these activity data were transformed into metabolic equivalent hours per week (METs/h/week)^41–43^.

### Genotyping

We selected 6 SNPs (rs1421085, rs1121980, rs17817449, rs8050136, rs9939973, and rs3751812) within the region of the *FTO* gene, based on published literature and on the validated catalog of published genome-wide association studies^44^ and the Phenotype-Genotype Integrator^45^, taking into account minor allele frequency (MAF) > 0.2 and P values < 10^−7^. The selected SNPs were associated with dietary intake or obesity phenotypes^46–51^. The linkage disequilibrium between rs9939609 and the three SNPs (rs8050136, rs3751812, rs17817449) is high (r^2^ = 1) based on data from South and East Asians, hence the rs9939609 was not entered in GRS calculation and analysis^7,52^. Also due to limited resources available, the most reproducible associated SNPs with obesity and dietary intake were selected.

Genomic DNA was extracted from peripheral blood using a standard Proteinase K, salting-out method^53^. Six SNPs were selected through the NCBI site. Our T-ARMS assay with different inner allele specific primers was used to produce allele-specific PCR products. The two outer primers produced a PCR product that was used as an internal control for the reaction. For all six SNPs, the PCR reaction (Thermal Cycler, Corbett Life Science, Sydney, Australia) was optimized in a 12.5 µl total volume containing 1.5 µl DNA template, 6.25 µl Master Mix containing MgCl_2_, Smart Taq polymerase (CinnaGene Co.; Tehran, Iran), and 0.1% BSA (TaKaRa; Kusatsu, Japan), 2 µl primer (outer and inner), and 2.75 µl water. The PCR products were separated by size by agarose gel electrophoresis, and each genotype generated a specific band. Accuracy of the results was confirmed by direct sequencing of 10% of each sample using the outer primers.

### Obesity GRS calculation

GRS was calculated based on the 6 SNPs using the weighted method^24,54^. Each SNP was recoded as 0, 1, or 2 according to the number of risk alleles^44,45^ (BMI increasing alleles), and each SNP was weighted by its relative effect size (odds ratio) derived from the previously reported meta-analysis or original data (Table 2). We then calculated the GRS using the following equation:

$$\mathrm{GRS}=(\mathrm{OR1}\times \mathrm{SNP1}+\mathrm{OR2}\times \mathrm{SNP2}+{\rm{\ldots }}+\mathrm{ORn}\times \mathrm{SNPn})\times ({\rm{n}}/\mathrm{sum\; of\; the\; ORs}),$$

where OR is the odds ratio of each individual SNP on BMI, as derived from the previous literature and reported meta-analysis^46,49,52^, n is 6, and sum of the ORs is 8.18 in the current analysis. The GRS ranged from 0 to 12, and each point of the GRS corresponded to each single risk allele.

### Definitions

Obesity was defined as a BMI ≥ 30 kg/m^2^, and a BMI between 18.5 and 24.9 classified a person as having a normal weight^55^. The WC > 95 cm for both genders^56^, as well as WHR > 0.8 in men and > 0.9 in women, were considered as indicators of abdominal obesity^57^.

### Statistical analysis

The descriptive analysis consisted of a comparison of qualitative and quantitative variables between cases and controls using the Chi square and Student t test, respectively; the genotype and allele frequencies for the analyzed polymorphisms were obtained using Power-Marker software. Pearson’s Chi-square statistic test was used to calculate the Hardy-Weinberg equilibrium.

Conditional logistic regression was used to estimate the interactions of SNPs and GRS (GRS ≥ 6, GRS < 6) with quartiles of dietary fiber intake (normal distribution) (Q1: < 7.06, Q2:7.06–9.13, Q3:9.14–11.26, Q4 > 11.26 g/4186 KJ) or high and low dietary fiber (≥14 and < 14 gram/gay)^58^ in relation to obesity, after adjustment for educational level (≤14 and > 14 years) and energy intake. Two likelihood scores were obtained by performing this statistical analysis, with and without the interaction terms; the P value for interaction was determined by performing the likelihood ratio test.

Conditional logistic regression was used to generate odds ratios (ORs) for obesity of individuals as carriers or non-carriers of risk alleles of each SNP across quartiles of dietary fiber intake. The highest quartile of dietary fiber intake and the homozygote group with a major allele were examined as the reference group. Participants were divided into two groups based on the median GRS. Unconditional logistic regression was performed to estimate the interactions of SNPs and GRS with quartiles of dietary fiber intake in relation to abdominal obesity. All ORs were adjusted for variables proven to be associated with obesity, including age, gender, educational level, smoking status (current, ex-smoker, or never smoked), physical activity (low, moderate, and high) and energy intake. The P value for trend across the quartiles of dietary fiber was determined using logistic regression, with the median of each quartile of dietary fiber intake as a continuous variable. Data were analyzed using the STATA statistical package v.12.0 or the Statistical Package for Social Sciences (Version 20.0; SPSS Inc., IBM, New York, NY, USA).

### Data availability

The datasets generated during and/or analyzed during the current study are available from the corresponding author on reasonable request.
